# A History of Large for Gestational Age at Birth and Future Risk for Pediatric Neoplasms: A Population-Based Cohort Study

**DOI:** 10.3390/jcm9051336

**Published:** 2020-05-04

**Authors:** Roy Kessous, Eyal Sheiner, Daniella Landau, Tamar Wainstock

**Affiliations:** 1 Department of Obstetrics and Gynecology, Soroka University Medical Center, Ben-Gurion University of the Negev, Beer-Sheva 84101, Israel; kessousr@bgu.ac.il; 2 Department of Neonatology, Soroka University Medical Center, Ben-Gurion University of the Negev, Beer-Sheva 84101, Israel; landaud@bgu.ac.il; 3 The Department of Public Health, Faculty of Health Sciences, Ben-Gurion University of the Negev, Beer-Sheva 84101, Israel; wainstoc@bgu.ac.il

**Keywords:** large for gestational age, childhood malignancy, leukemia, kidney tumors

## Abstract

Objective: The aim of this study was to evaluate the association between large for gestational age (LGA) at birth and future risk of childhood neoplasm. Study design: a population-based cohort to compare the long-term risk (up to the age of 18 years) of childhood neoplasms (benign and malignant) in children that were born LGA vs. those that were appropriate for gestational age (AGA), between the years 1991–2014. Childhood neoplasms diagnosis were defined according to international classification of disease 9 (ICD-9) codes recorded medical files. Kaplan–Meier survival curves were used in order to compare cumulative incidence of oncological morbidity over the study period. The Cox proportional hazards model was used to control for confounders. Results: 231,344 infants met the inclusion criteria; out of those 10,369 were diagnosed LGA at birth. Children that were LGA at birth had a higher incidence of leukemia (OR 2.25, 95%CI 1.08–4.65, *p* = 0.025) as well as kidney tumors (OR = 4.7, 95%CI = 1.02–21.9, *p* = 0.028). In addition, cumulative incidence over time of childhood malignancies, leukemia, and kidney tumors were significantly higher in LGA children (Log Rank = 0.010, 0.021, and 0.028, respectively). In a Cox regression model controlling for other perinatal confounders, LGA at birth remained independently associated with an increased risk for childhood malignancy (adjusted HR 1.51, 95%CI 1.02–2.23, *p* = 0.039). Conclusion: LGA at birth is associated with increased long-term risk for childhood malignancy and specifically leukemia and kidney tumors. This possible link may help to improve current knowledge regarding potential exposures that are associated with childhood cancer development.

## 1. Introduction

The theory of fetal “programing” during pregnancy deals with the possibility that some intrauterine exposures cause changes in fetal development that may have implementations on future risk for specific diseases during childhood [1]. Several mechanisms for this theory have been proposed including the influence on cell division during the early embryonic phase that leads to epigenetic changes [2]. In addition, hormonal imbalances such as in women with polycystic ovary syndrome, are known to create a sub-optimal intrauterine environment that may lead to genetic alternations and increased future risk for several comorbidities in the offspring [3].

In general, the birth weight of children in Israel has been shown to be slightly lower that of children born in European and North American countries but higher than that of black Americans and Japanese [4]. Large for gestational age (LGA) is defined as birth weight greater than that of the 90th or 95th percentile adjusted for gestational age and fetal gender [5,6]. These definitions are accepted worldwide and also used by the Israel Society of Obstetrics and Gynecology and have not changed in the last two decades [4]. The short-term complications related to LGA are well known and reported extensively in the literature [7]. Recently, there is growing interest in literature regarding the possible long-term association between intrauterine environmental exposures and future health implications on the offspring during childhood [8].

With regard to the association between LGA and the future risk for childhood malignancies, data in the literature is not abundant and sometimes contradicting. Results from previous studies generally support this association with published data that show an increased risk for childhood cancer and specifically malignancies, such as leukemia and central nervous system tumors in children who were born LGA [9,10,11,12,13,14]. In a study by Paltiel et al. authors found a linear relation between increasing birth weight (each kg) and all types of childhood cancer and specifically leukemia [9]. Another study looked specifically on the incidence of acute lymphoblastic leukemia and found similar linear correlation with increased birth weight [10]. Nevertheless, despite this trend in literature, other studies have failed to show significant positive association between birth weight and future childhood malignancy incidence [15,16]. Given the nature of the tested association as well as methodological difficulties that exist in various studies, further validation for the association is needed. Cancer is a major cause of death in children, and several childhood malignancies have early onset in nature with little knowledge regarding the underlining causes [17]. Given the immense implications of childhood cancers as well as contradicting results in previously published studies, adding information regarding this possible association between LGA and childhood malignancies is of great importance in order to evaluate possible risk factors for cancer development during childhood. Hence, the aim of this study was to use two independent databases in order to evaluate the association between a history of LGA at birth and future risk of childhood neoplasms.

## 2. Materials and Methods

### 2.1. Study Population

A population-based retrospective cohort study that included all the deliveries (between the years 1991–2014) at the Soroka University Medical Center (SUMC), the only tertiary center in southern Israel. Data analysis in the study is based on a non-selective population. The study was approved by the institutional review board (SUMC IRB committee, approval number 0220-17-SOR).

### 2.2. Study Design

Long-term incidence (up to the age of 18) of childhood neoplasms was compared between children who were born with the diagnosis of LGA (birth weight ≥ 95th centile adjusted for gestational age and fetal gender in accordance with the World Health Organization definitions) [18] and those who were born appropriate for gestational age (AGA) (5th centile  < birth weight < 95th centile). We excluded from the analysis multiple gestations, children that were diagnosed as small for gestational age as well as those that were diagnosed with congenital malformations. We collected and assessed maternal demographic and pregnancy characteristics as well as delivery outcome and adverse perinatal outcomes. The measured outcomes were long-term childhood neoplasm related up to the age of 18 years. A list of childhood neoplasms diagnoses based on international classification of disease 9 (ICD-9) code was predefined and used for the collection of outcomes (Table S1). Children were followed up to the first hospitalization related to one of the listed neoplasms (recurrent hospitalizations were not considered), or in case of a hospitalization that resulted in death (not malignancy-related), or when the child reached the age of 18 years.

The collection of data was performed using two databases, one was the SUMC (“Demog-ICD9”) computerized hospitalization database and the second was a computerized perinatal database from the obstetrics and gynecology department. The data was cross-linked and merged for the purpose of data analysis. The hospitalization database comprises all demographic information as well as medical diagnosis (coded to ICD-9 codes) that were made during hospitalizations at SUMC. ICD-9 coding information in our institution provides coding according to the topography as well as the behavior of a neoplasm; however, it does not provide information regarding the morphology of malignancies. Perinatal data is recorded following each delivery by an obstetrician and is reviewed and recorded to the database by experienced medical secretaries routinely. This process of collecting data is assuring maximal completeness and accuracy of the information collected.

### 2.3. Statistical Analysis

In order to perform the statistical analysis, we used the SPSS package 23rd ed. (SPSS, Chicago, IL, USA). Chi-square was used to compare general associations between categorical data. Differences in continuous variables were assessed using the Student t-test. Kaplan–Meier curves for survival were used in order to compare the incidence of neoplasms throughout the study period. Log-rank test was used to assess differences between children that were born LGA to those that were AGA at birth.

In order to establish independent association and control for other confounding variables we used the Cox proportional hazards model analysis. The potential confounders that were evaluated included maternal age at delivery, gestational age at delivery, gestational diabetes mellitus, and pre-pregnancy obesity. All analyses were two-sided, and a *p*-value of < 0.05 was considered statistically significant.

## 3. Results

231,344 infants met the inclusion criteria during the study period; out of those 10,369 were diagnosed as LGA at birth. In our cohort, the 95th percentile for the whole cohort (not adjusted for gestational age) for boys and girls were 4.05 kg and 3.9 kg, respectively. These values are comparable to the crude values according to the WHO (boys 4.2 kg and girls 4.0 kg). Table 1 presents maternal and fetal characteristics and perinatal outcome for the study groups. Mothers to infants that were born LGA were older and had higher incidence of pre-pregnancy obesity, hypertension disorders, diabetes mellitus (both pre-gestational and gestational), and cesarean delivery. The mean birth weight at the LGA group was 4174 g, and the mean follow up time was ten years.

Table 2 presents the incidence of long-term childhood neoplasms related hospitalization. Children that were LGA at birth had significantly increased risk for childhood leukemia compared to those that were AGA at birth. In addition, children that were LGA had significantly higher incidence of kidney neoplasm as well as a trend (not statistically significant) for higher incidence of brain tumors. The incidence of benign neoplasms was not significantly different between the two groups (OR 1.2, 95%CI 0.93–1.60; *p* value = 0.140). However, the incidence of total neoplasm related hospitalizations as well as malignancy related hospitalizations were significantly higher in the LGA group (OR 1.6, 95%CI 1.09–2.35; *p* value = 0.015 and OR 1.3, 95%CI 1.07–1.66; *p* value = 0.010; respectively).

There were no differences between the fetal gender in all neoplasm morbidities (rates in males were 48.5% versus 50.8% among offspring diagnosed and not diagnosed with neoplasm morbidities, respectively, *p* = 0.09) and all-cancer morbidities (rates of males were 53.9% and 50.8% among cancer and non-cancer cases, respectively, *p* = 0.23).

With regard to possible differences in age of diagnosis, we have compared data regarding median age at diagnosis (between LGA and AGA) for benign, malignant, and total neoplasm rate. Median age at diagnosis did not differ between the groups for any of the categories (*p* value = 0.86, 0.88, and 0.43, respectively).

**Figure 1:**
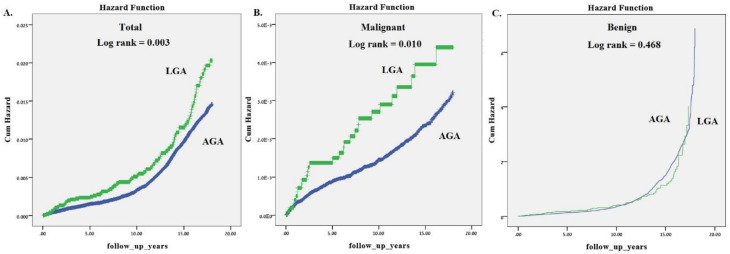
Cumulative childhood malignant tumors according to whether there were LGA or not during index pregnancy. (**A**) Total neoplasm related hospitalizations, (**B**) childhood malignancy hospitalizations only, and (**C**) benign related hospitalizations only.

**Figure 2:**
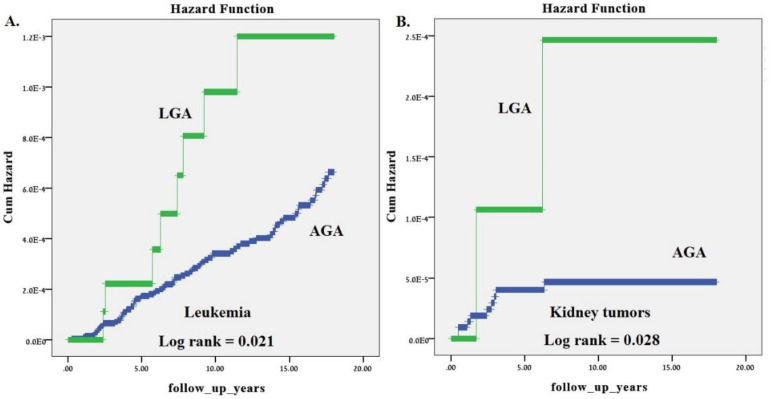
Cumulative childhood related hospitalizations due to malignant tumors according to whether there were LGA or not and the type of tumor. (**A**) Leukemia and (**B**) kidney tumors.

In order to evaluate cumulative incidence of childhood neoplasms during the study period we used Kaplan–Meier survival curves total incidence as well as malignant and benign (Figure 1). Children that were LGA at birth had significantly higher incidence of total childhood neoplasms (Log Rank *p* value of 0.003) as well as childhood malignancies (Log Rank *p* value of 0.010) compared to those that were born AGA. Figure 2 demonstrates the higher long-term cumulative incidence of childhood leukemia (A) and kidney tumors (B) in children that were born LGA compared to those that were AGA at birth (Log Rank *p* value of 0.021 and 0.028, respectively).

The Cox proportional hazards model for assessing the risk of childhood malignancies in children that were born with a diagnosis of LGA while controlling for confounders is presented in Table 3. The confounders that were controlled included variables that were significantly different between the studied groups in the univariable analysis. Those included maternal age, diabetes mellitus (both pre-gestational and gestational), hypertensive disorders, cesarean section, pre-pregnancy obesity, and gestational age at delivery. After controlling for confounders, LGA at birth remained independently associated with an increased risk for childhood neoplasms (adjusted HR 1.51, 95%CI 1.02–2.23, *p* = 0.039).

## 4. Discussion

In the current study we found that a diagnosis of LGA at birth is significantly related to increased incidence of total malignant neoplasms during childhood, specifically leukemia and kidney cancer. The results of our study are adding to previously published studies from more than seven geographical areas in the world showing similar relations and a linear relation between increasing birth weight and childhood cancer [9,11]. Malignancy is one of the major causes of death during childhood [17,19]. Scarce data exists regarding the etiology of childhood malignancies and only a small number of risk factors have been established [20]. Nonetheless, their typical early age presentation as well as previously published data that indicated that in pediatric leukemia the majority of chromosome translocations arises in utero, suggest that prenatal influences on cancer development may exist [21]. Therefore, the addition of data that might help confirm known associations or find out about new potential risk factors for the development of childhood malignancies is of great significance.

### 4.1. Leukemia

Several previous case control studies have tried to evaluate the association between birth weight and future risk for leukemia [12,14,22,23]. In a study by Da Silva et al. authors evaluated the incidence of acute lymphoblastic leukemia with relation to birth weight and found a linear increase in the incidence of childhood leukemia with each 1000 g increase in birth weight [10]. Schuz et al. performed a population-based control study and used parents’ questionnaire reported risk factors and found that high birth weight (>4 kg) is significantly associated with leukemia during childhood (OR = 1.41) [22]. In our study we used a perinatal electronic database that reduces the risk for recall bias. Three other studies have reported results of case control studies that are based on birth registries. All of these studies have matched controls by age and some by other factors such as gestational age. The results of all three studies show significant association between high birth weight and childhood leukemia (OR = 1.1 [23], OR = 1.3 [14], and OR = 1.1 [12]). In our study we performed a population-based study using the registry of the single tertiary hospital in our region in order to perform a long follow-up of children using the pediatric database. The childhood leukemia rate in the AGA group in the current study matched the expected rates reported in the Israeli national cancer registry [24]. Using this information, we found that children that were LGA at birth have significantly increased risk for childhood leukemia compared to AGA children (OR 2.25, 95%CI 1.08–4.65, *p* = 0.025). When considering the results of the current study together with previously published studies, there seems to be a link between a history of LGA at birth and future risk for childhood leukemia. Current information may lead to further studies evaluating this association.

### 4.2. Kidney Tumors

Two previous studies have evaluated the association between birth weight and future risk for kidney tumors. In a study by O’Neill et al. authors reported data that was gathered from registries from both the United States (US) and the United Kingdom (UK) [25]. In this study, the total cancer risk increased with each 0.5 kg increase in birthweight. More specifically, authors found that children who were LGA at birth had an increased risk for renal tumors, both in the US and UK populations (OR = 1.17, 95%CI = 1.10–1.24 and OR = 1.12, 95%CI = 1.06–1.19, respectively) [12]. Moreover, similar results were reported in a study that analyzed registries of several Nordic countries; results from this study show a significant increase in the incidence of Wilm’s tumor in children who were born LGA (OR = 2.1, 95%CI = 1.2–3.6) [14]. In the current study, we found a significantly increased risk for the development of kidney tumors in children that were born LGA (OR = 4.7, 95%CI = 1.02–21.9, *p* = 0.028). Taking together the results of the current study and previously published studies, despite the relatively small number of studies and their limitations, we can conclude that a positive association probably exists between LGA and kidney tumors.

### 4.3. Brain Tumors

Controversy exists regarding the association between birth weight and brain tumors. Several previous studies have showed higher incidence of brain tumors in children who had a history of LGA at birth [12,13]. However, contradicting results were found in several other studies; an Australian study by Greenop et al. found no association between birth characteristics including birth weight and future risk for childhood brain tumor [16]. Another study from France evaluated 510 cases and 3102 matched control and also found no association between birth weight and the risk for brain tumors during childhood (OR = 0.8, 95%CI = 0.5–1.4) [15]. Finally, Bjørge et al. found a significant association between absolute birth weight above 4 kg and future childhood CNS tumors, but when they analyzed the risk for LGA, this association was not statistically significant (OR = 1.1, 95%CI = 0.85–1.4) [14]. In our study, an increased incidence of brain tumors was found in children with a history of LGA at birth, but this association was not statistically significant (OR = 2.0, 95%CI = 0.61–6.5). This difficulty in establishing an association between LGA and brain tumors probably lies in the fact that these tumors are relatively rare and so perhaps this is the place were meta-analysis of existing data is in order. Recently, Georgakis et al. published a systematic meta-analysis that included results from forty-one articles, in this study authors reported that children with a history of LGA at birth are at increased risk for future central nervous system (CNS) tumors (OR = 1.12, 95%CI = 1.03–1.22) [26]. Despite the inconsistency in reported studies, it seems that possible association may exist between LGA and future risk for childhood brain tumors. Further studies are needed in order to confirm this association.

The strengths of the current study lie in the low probability for incorrect outcome measurements as well as recall bias since it is based on results that were collected from a computerized database of a single regional medical center. Virtually all the deliveries that are taking place in our region are managed in our hospital, and this same center is also providing comprehensive pediatric care for children in our region. This has enabled us to assess the association of perinatal characteristics, such as LGA, and a relatively long-term outcome such as childhood malignancy while controlling many confounding parameters. There are also limitations for the current study; our major limitation comes from the fact that childhood malignancy incidence is relatively low and the absolute case number for each specific malignancy is small. Nevertheless, we were able to show significant differences in specific cancer types as well as in the total childhood malignancy incidence. Moreover, conclusions regarding specific types of cancer should be carefully made and based on the sum of results from the different studies that were published. In our hospitalization database, coding of malignancy diagnosis is based on ICD-9 coding that relates to the topography as well as the behavior of a neoplasm. Unfortunately, the data do not provide information regarding histologic sub-types (ICD-O-3 coding). Finally, our database does not include data regarding other possible exposures such as environmental exposures that can perhaps be associated with childhood cancer risk. Nonetheless, in this study, the population came from the same region and it is reasonable to assume that these exposures were similar between children with and without a history of LGA at birth.

In conclusion, in the current study we found a significant association between a history of LGA at birth and the future risk for childhood malignancy, specifically leukemia and kidney tumors. Cancer is one of the leading causes of death during childhood and our knowledge of the underlining causes and risk factors is very limited. Additional studies are needed in order to improve current knowledge regarding potential exposures that are associated with childhood cancer development.

## Figures and Tables

**Table 1:** Maternal demographics and pregnancy characteristics at index pregnancy divided into patients with and without a history of large for gestational age (LGA).

Characteristic		LGA *n* = 10,369 (%)	AGA *n* = 220,975 (%)	*p* Value
Maternal age (years ± SD)		30.7 ± 5.6	28.1 ± 5.8	0.019
Gestational age in weeks (mean ± SD)		39.1 ± 1.8	39.1 ± 1.9	0.119
Pre-pregnancy obesity (BMI >= 30)		2.9%	1.0%	0.001
Cesarean section		26.5%	12.7%	0.001
Hypertension (Pre-gestational and gestational)		6.4%	4.8%	0.001
Pre-gestational and gestational diabetes mellitus		15.3%	4.6%	0.001
Fetus gender	male	49.9%	50.8%	0.066
	female	50.1%	49.2%	
Mean birth weight (g)		4174 ± 509	3207 ± 420	0.001
Mean follow up time (years)		10.0 ± 5.8	10.3 ± 5.9	0.001

The data are presented as % (*n*) or mean ± SD; the significance for differences was measured using the Chi squared and Mann–Whitney test or Student *t*-test.

**Table 2:** The childhood malignancy rate (per 1000) following maternal history of LGA in index pregnancy vs. no LGA.

	LGA *n* = 10,369 (Per 1000 Person Years)	No LGA *n* = 220,975 (Per 1000 Person Years)	Hazard Ratio; 95% CI	*p* Value
Head and Neck (*n* = 12)	0 (-)	12 (0.005)	-	0.453
Lung (*n* = 3)	0 (-)	3 (0.001)	-	0.708
Bone (*n* = 12)	0 (-)	12 (0.005)	-	0.453
Skin (*n* = 4)	1 (0.009)	3 (0.001)	7.34; 0.76–70.0	0.084
Kidney (*n* = 11)	2 (0.019)	9 (0.004)	4.74; 1.02–21.95	0.046
Brain (*n* = 35)	3 (0.028)	32(0.014)	2.05; 0.63–6.70	0.234
Ophthalmic (*n* = 4)	0 (-)	4 (0.002)	-	0.665
connective tissue (*n* = 12)	0 (-)	12 (0.005)	-	0.453
Lymphoma (*n* = 49)	3 (0.028)	46 (0.020)	1.44; 0.45–4.64	0.538
Leukemia (*n* = 84)	8 (0.076)	76 (0.033)	2.31; 1.14–4.78	0.024
Benign (*n* = 1031)	56 (0.536)	975 (0.428)	1.30; 0.99–1.70	0.056
Malignant (*n* = 401)	28 (0.268)	373 (0.164)	1.65; 1.12–2.42	0.011
Total oncological hospitalizations (*n* = 1428)	84 (0.905)	1344 (0.591)	1.40; 1.12–1.75	0.003

**Table 3:** The Cox multivariable regression model to evaluate the risk of childhood malignancies in children who were born LGA while controlling for confounders.

	Adjusted HR	CI 95%	*p* Value
LGA	1.51	1.02–2.23	0.039
Gestational diabetes mellitus	1.36	0.94–1.97	0.102
Maternal age at index birth	0.99	0.97–1.02	0.930
Hypertensive disorders	0.76	0.43–1.15	0.162
Cesarean section	1.46	1.12–1.92	0.005
Pre-pregnancy obesity	1.08	0.48–2.47	0.858
Gestational age at birth	0.97	0.92–1.02	0.290
Fetal gender	1.12	0.91–1.36	0.267

## Supplementary Materials

The following are available online at https://www.mdpi.com/2077-0383/9/5/1336/s1, Table S1: List of childhood neoplasms diagnoses based on international classification of disease 9 (ICD-9) code.
