# Supplementary material for: A History of Large for Gestational Age at Birth and Future Risk for Pediatric Neoplasms: A Population-Based Cohort Study
Source: J Clin Med. 2020 May 4;9(5):1336. doi: 10.3390/jcm9051336 (PMC7291000; doi:10.3390/jcm9051336)
Supplement: Supplementary file 1 [file jcm-09-01336-s001.pdf]

Table 1. list of childhood neoplasms diagnoses based on international classification of disease 9 (ICD-9) code.

**Diagnosis Code**	Diagnosis	Subgroup
**193**	MALIGNANT NEOPLASM OF THYROID GLAND	head and neck
**1409**	MALIGNANT NEOPLASM OF LIP, UNSP., VERMILION BORDER	head and neck
**1420**	MALIGNANT NEOPLASM OF PAROTID GLAND	head and neck
**1450**	MALIGNANT NEOPLASM OF CHEEK MUCOSA	head and neck
**1479**	MALIGNANT NEOPLASM OF NASOPHARYNX, UNSPECIFIED	head and neck
**1950**	MALIGNANT NEOPLASM OF HEAD, FACE, AND NECK	head and neck
**1502**	MALIGNANT NEOPLASM OF ABDOMINAL ESOPHAGUS	esophagus
**1519**	MALIGNANT NEOPLASM OF STOMACH, UNSPECIFIED	stomach
**1540**	MALIGNANT NEOPLASM OF RECTOSIGMOID JUNCTION	colon rectum
**1541**	MALIGNANT NEOPLASM OF RECTUM	colon rectum
**V1006**	PERSONAL HISTORY OF MAL.NEO.RECTUM,RECTOSIGMOID JUNCTION,ANUS	colon rectum
**V1009**	PERSONAL HISTORY MAL.NEOPL.OTHER SITES IN GASTROINTEST.TRACT	colon rectum
**1550**	MALIGNANT NEOPLASM OF LIVER, PRIMARY	liver
**1570**	MALIGNANT NEOPLASM OF HEAD OF PANCREAS	pancreas
**1579**	MALIGNANT NEOPLASM OF PANCREAS, PART UNSPECIFIED	pancreas
**1580**	MALIGNANT NEOPLASM OF RETROPERITONEUM	retroperitoneum
**1589**	MALIGNANT NEOPLASM OF PERITONEUM, UNSPECIFIED	peritoneum
**1629**	MALIGNANT NEOPLASM OF BRONCHUS AND LUNG, UNSPECIFIED	lung
**2319**	CARCINOMA IN SITU OF RESPIRATORY SYSTEM, PART UNSPECIFIED	lung
**2391**	NEOPLASM OF UNSPECIFIED NATURE OF RESPIRATORY SYSTEM	lung
**V102**	PERSONAL HISTORY MAL.NEOPL.OTHER RESPIR.AND INTRATHOR.ORGANS	lung
**1642**	MALIGNANT NEOPLASM OF ANTERIOR MEDIASTINUM	mediastinum
**1649**	MALIGNANT NEOPLASM OF MEDIASTINUM, PART UNSPECIFIED	mediastinum
**1701**	MALIGNANT NEOPLASM OF MANDIBLE	bone

**1702**	MAL.NEOPLASM OF VERTEBRAL COLUMN, EXCLUDING SACRUM & COCCYX	bone
**1703**	MALIGNANT NEOPLASM OF RIBS, STERNUM, AND CLAVICLE	bone
**1704**	MALIGNANT NEOPLASM OF SCAPULA AND LONG BONES OF UPPER LIMB	bone
**1706**	MALIGNANT NEOPLASM OF PELVIC BONES, SACRUM, AND COCCYX	bone
**1707**	MALIGNANT NEOPLASM OF LONG BONES OF LOWER LIMB	bone
**1708**	MALIGNANT NEOPLASM OF SHORT BONES OF LOWER LIMB	bone
**1709**	MAL.NEOPLASM OF BONE AND ARTICULAR CARTILAGE, SITE UNSPEC.	bone
**V1081**	PERSONAL HISTORY OF MALIGNANT NEOPLASM OF BONE	bone
**1710**	MAL.NEOPLASM OF CONNECTIVE AND SOFT TISSUE,HEAD,FACE,NECK	connective tissue
**1712**	MAL.NEOPLASM CONNEC.AND SOFT TISSUE,UPPER LIMB,INCL.SHOULDER	connective tissue
**1713**	MAL.NEOPLASM OF CONNECTIVE AND SOFT TISSUE LOWER LIMB,INCL.HIP	connective tissue
**1714**	MAL.NEOPLASM OF CONNECTIVE AND OTHER SOFT TISSUE OF THORAX	connective tissue
**1715**	MAL.NEOPLASM OF CONNECTIVE AND OTHER SOFT TISSUE OF ABDOMEN	connective tissue
**1716**	MAL.NEOPLASM OF CONNECTIVE AND OTHER SOFT TISSUE OF PELVIS	connective tissue
**1717**	MAL.NEOPLASM OF CONNECTIVE AND SOFT TISSUE OF TRUNK,UNSPECIFIED	connective tissue
**1718**	MAL.NEO.OF OTHER SPEC. SITES OF CONNECT.AND SOFT TISSUE	connective tissue
**1719**	MAL.NEOPLASM OF CONNECTIVE AND SOFT TISSUE,SITE UNSPECIFIED	connective tissue
**173**	OTHER MALIGNANT NEOPLASM OF SKIN	skin
**1725**	MALIGNANT MELANOMA OF SKIN OF TRUNK, EXCEPT SCROTUM	skin
**1726**	MALIGNANT MELANOMA OF SKIN OF UPPER LIMB, INCLUDING SHOULDER	skin
**1729**	MELANOMA OF SKIN, SITE UNSPECIFIED	skin
**1733**	MALIGNANT NEOPLASM OF SKIN OF UNSPECIFIED PARTS OF FACE	skin
**V1082**	PERSONAL HISTORY OF MALIGNANT MELANOMA OF SKIN	skin
**1741**	MALIGNANT NEOPLASM OF CENTRAL PORTION OF FEMALE BREAST	breast
**1830**	MALIGNANT NEOPLASM OF OVARY	ovary
**1840**	MALIGNANT NEOPLASM OF VAGINA	vagina vulva
**1844**	MALIGNANT NEOPLASM OF VULVA, UNSPECIFIED	vagina vulva
**2123**	BENIGN NEOPLASM OF BRONCHUS AND LUNG	vagina vulva
**2462**	CYST OF THYROID	vagina vulva

**20960**	BENIGN CARCINOID TUMOR OF UNKNOWN PRIMARY SITE	vagina vulva
**1869**	MALIGNANT NEOPLASM OF OTHER AND UNSPECIFIED TESTIS	testis
**V1047**	PERSONAL HISTORY OF MALIGNANT NEOPLASM OF TESTIS	testis
**1889**	MALIGNANT NEOPLASM OF BLADDER, PART UNSPECIFIED	bladder
**1890**	MALIGNANT NEOPLASM OF KIDNEY, EXCEPT PELVIS	kidney
**V1052**	PERSONAL HISTORY OF MALIGNANT NEOPLASM OF KIDNEY	kidney
**1905**	MALIGNANT NEOPLASM OF RETINA	ophthalmic
**V1084**	PERSONAL HISTORY OF MALIGNANT NEOPLASM OF EYE	ophthalmic
**1910**	MALIGNANT NEOPLASM OF CEREBRUM, EXCEPT LOBES AND VENTRICLES	brain
**1912**	MALIGNANT NEOPLASM OF TEMPORAL LOBE	brain
**1913**	MALIGNANT NEOPLASM OF PARIETAL LOBE	brain
**1914**	MALIGNANT NEOPLASM OF OCCIPITAL LOBE	brain
**1915**	MALIGNANT NEOPLASM OF VENTRICLES	brain
**1916**	MALIGNANT NEOPLASM OF CEREBELLUM NOS	brain
**1917**	MALIGNANT NEOPLASM OF BRAIN STEM	brain
**1918**	MALIGNANT NEOPLASM OF OTHER PARTS OF BRAIN	brain
**1919**	MALIGNANT NEOPLASM OF BRAIN, UNSPECIFIED	brain
**1920**	MALIGNANT NEOPLASM OF CRANIAL NERVES	brain
**1921**	MALIGNANT NEOPLASM OF CEREBRAL MENINGES	brain
**1928**	MALIGNANT NEOPLASM OF OTHER SPECIFIED SITES OF NERVOUS SYSTEM	brain
**V1085**	PERSONAL HISTORY OF MALIGNANT NEOPLASM OF BRAIN	brain
**1940**	MALIGNANT NEOPLASM OF ADRENAL GLAND	adrenal
**1960**	2ND AND UNSP.MAL.NEOPL.OF LYMPH NODES,HEAD,FACE AND NECK	secondary
**1963**	2ND AND UNSP.MAL.NEOPL.OF LYMPH NODES OF AXILLA+UPPER LIMB	secondary
**1970**	SECONDARY MALIGNANT NEOPLASM OF LUNG	secondary
**1980**	SECONDARY MALIGNANT NEOPLASM OF KIDNEY	secondary
**1983**	SECONDARY MALIGNANT NEOPLASM OF BRAIN AND SPINAL CORD	secondary
**1985**	SECONDARY MALIGNANT NEOPLASM OF BONE AND BONE MARROW	secondary
**19889**	SECONDARY MALIGNANT NEOPLASM OF OTHER SPECIFIED SITES	secondary

**202**	OTHER MALIGNANT NEOPLASMS OF LYMPHOID & HISTIOCYTIC TISSUE	lymphoma
**2001**	LYMPHOSARCOMA	lymphoma
**2002**	BURKITT'S TUMOR OR LYMPHOMA	lymphoma
**2021**	MYCOSIS FUNGOIDES	lymphoma
**2028**	OTHER MALIGNANT LYMPHOMAS	lymphoma
**20011**	LYMPHOSARCOMA INVOLVING LYMPH NODES OF HEAD, FACE, AND NECK	lymphoma
**20012**	LYMPHOSARCOMA INVOLVING INTRATHORACIC LYMPH NODES	lymphoma
**20013**	LYMPHOSARCOMA INVOLVING INTRA-ABDOMINAL LYMPH NODES	lymphoma
**20020**	BURKITT'S TUMOR OR LYMPHOMA, UNSP.SITE, EXTRANODAL & SOLID ORGAN SITES	lymphoma
**20021**	BURKITT'S TUMOR;LYMPHOMA INVOLV.LYMPH NODES HEAD,FACE,NECK	lymphoma
**20023**	BURKITT'S TUMOR,LYMPHOMA INVOLV.INTRA-ABDOMINAL LYMPH NODES	lymphoma
**20025**	BURKITT'S TUMOR;LYMPHOMA INV.LYMPH NODES INGUIN.REG./LOWER LIMB	lymphoma
**20028**	BURKITT'S TUMOR;LYMPHOMA INVOLVING LYMPH NODES MULTIPLE SITES	lymphoma
**20151**	HODGKIN'S DIS.NODULAR SCLEROSIS,INVOLVING HEAD,FACE,NECK	lymphoma
**20152**	HODGKIN'S DIS.NODULAR SCLEROSIS,INVOLV.INTRATHORACIC LYMPH N.	lymphoma
**20161**	HODGKIN'S DIS.MIXED CELLULARITY,INVOLVOLVING HEAD,FACE,NECK	lymphoma
**20162**	HODGKIN'S DIS.MIXED CELLULARITY,INVOLV.INTRATORACIC LYMPH N.	lymphoma
**20190**	HODGKIN'S DIS.,UNSP. TYPE, UNSP. SITE, EXTRANODAL & SOLID ORGAN SITES	lymphoma
**20191**	HODGKIN'S DIS.UNSPECIFIED TYPE,INVOLVING HEAD,FACE,NECK	lymphoma
**20192**	HODGKIN'S DISEASE UNSP.TYPE,INVOLV.INTRATHORACIC LYMPH NODES	lymphoma
**20193**	HODGKIN'S DISEASE UNSP.TYPE,INVOLV.INTRA-ABDOMINAL LYMPH NODES	lymphoma
**20196**	HODGKIN'S DIS.UNSPECIFIED TYPE,INVOLV.INTRAPELVIC LYMPH NODES	lymphoma
**20198**	HODGKIN'S DIS.UNSPECIFIED TYPE,INVOLVING MULTIPLE SITES	lymphoma
**20200**	NODULAR LYMPHOMA, UNSP.SITE, EXTRANODAL & SOLID ORGAN SITES	lymphoma
**20202**	NODULAR LYMPHOMA INVOLVING INTRATHORACIC LYMPH NODES	lymphoma
**20210**	MYCOSIS FUNGOIDES, UNSP.SITE, EXTRANODL AND SOLID ORGAN SITES	lymphoma
**20211**	MYCOSIS FUNGOIDES INVOLVING LYMPH NODES OF HEAD, FACE, AND NECK	lymphoma
**20212**	MYCOSIS FUNGOIDES INVOLVING INTRATHORACIC LYMPH NODES	lymphoma
**20213**	MYCOSIS FUNGOIDES INVOLVING INTRA-ABDOMINAL LYMPH NODES	lymphoma

**20280**	OTHER MALIGNANT LYMPHOMAS, UNSP.SITE, EXTRANODAL & SOLID ORGAN SITES	lymphoma
**20281**	OTHER MALIGNANT LYMPHOMAS INVOLV.LYMPH NODES HEAD,FACE,NECK	lymphoma
**20282**	OTHER MALIGNANT LYMPHOMAS INVOLVING INTRATHORACIC LYMPH NODES	lymphoma
**20283**	OTHER MALIGNANT LYMPHOMAS INVOLVING INTRA-ABDOMINAL LYMPH NODES	lymphoma
**20285**	OTHER MALIGNANT LYMPHOMAS INVOLVING INGUINAL REG./LOWER LIMB	lymphoma
**20293**	UNSP.MAL.NEO.OF LYMPHOID/HISTIOCYTIC,INTRA-ABDOMINAL LYMPH N.	lymphoma
**V1071**	PERSONAL HISTORY OF LYMPHOSARCOMA AND RETICULOSARCOMA	lymphoma
**V1072**	PERSONAL HISTORY OF HODGKIN'S DISEASE	lymphoma
**V1079**	PERSONAL HISTORY OF OTHER LYMPHATIC + HEMATOPOIETIC NEOPL.	lymphoma
**204**	LYMPHOID LEUKEMIA	leukemia
**2040**	LYMPHOID LEUKEMIA, ACUTE	leukemia
**2040**	LYMPHOID LEUKEMIA, ACUTE__ 1992 פרט	leukemia
**2080**	LEUKEMIA OF UNSPECIFIED CELL TYPE, ACUTE__1992 פרט	leukemia
**2089**	UNSPECIFIED LEUKEMIA___1992 פרט	leukemia
**20300**	MULTIPLE MYELOMA WITHOUT MENTION OF REMISSION	leukemia
**20400**	ACUTE LYMPHOID LEUKEMIA WITHOUT MENT.OF HAVING ACHIEVED REMISSION	leukemia
**20400**	LYMPHOID LEUKEMIA, AC., WITHOUT MENTION OF REMISSION	leukemia
**20401**	LYMPHOID LEUKEMIA, ACUTE, IN REMISSION	leukemia
**20402**	ACUTE LYMPHOID LEUKEMIA IN RELAPSE	leukemia
**20500**	MYELOID LEUKEMIA, AC., WITHOUT MENTION OF HAVING ACHIEVED REMISS.	leukemia
**20500**	MYELOID LEUKEMIA, AC., WITHOUT MENTION OF REMISSION	leukemia
**20502**	ACUTE MYELOID LEUKEMIA IN RELAPSE	leukemia
**20510**	MYELOID LEUKEMIA, CHR., WITHOUT MENTION OF HAVING ACHIEVED REMIS.	leukemia
**20530**	MYELOID SARCOMA WITHOUT MENTION OF REMISSION	leukemia
**20720**	MEGAKARYOCYTIC LEUKEMIA WITHOUT MENTION OF REMISSION	leukemia
**20800**	AC.LEUKEMIA OF UNSP.CELL TYPE WITHO.MENT.OF HAVING ACHIEVED REMI.	leukemia
**20800**	LEUKEMIA, UNSP. CELL TYPE, AC., WITHOUT MENTION OF REMISSION	leukemia
**20801**	LEUKEMIA OF UNSP. CELL TYPE, AC., IN REMISSION	leukemia
**20890**	UNSP. LEUKEMIA WITHOUT MENTION OF HAVING ACHIEVED REMISSION	leukemia

**20890**	UNSP. LEUKEMIA WITHOUT REMISSION	leukemia
**27788**	TUMOR LYSIS SYNDROME	leukemia
**28522**	ANEMIA IN NEOPLASTIC DISEASE	leukemia
**V1061**	PERSONAL HISTORY OF LYMPHOID LEUKEMIA	leukemia
**V1062**	PERSONAL HISTORY OF MYELOID LEUKEMIA	leukemia
**V1069**	PERSONAL HISTORY OF OTHER LEUKEMIA	leukemia
**1735**	OTHER MALIGNANT NEOPLASM OF SKIN OF TRUNK, EXCEPT SCROTUM	OTHER
**1952**	MALIGNANT NEOPLASM OF ABDOMEN	OTHER
**2390**	NEOPLASM OF UNSPECIFIED NATURE OF DIGESTIVE SYSTEM	OTHER
**2392**	NEOPLASM OF UNSPECIFIED NATURE OF BONE, SOFT TISSUE, AND SKIN	OTHER
**2395**	NEOPLASM OF UNSPECIFIED NATURE OF OTHER GENITOURINARY ORGANS	OTHER
**2396**	NEOPLASM OF UNSPECIFIED NATURE OF BRAIN	OTHER
**2398**	NEOPLASM OF UNSPECIFIED NATURE OF OTHER SPECIFIED SITES	OTHER
**20002**	RETICULOSARCOMA INVOLVING INTRATHORACIC LYMPH NODES	OTHER
**23989**	NEOPLASM OF UNSPECIFIED NATURE OF OTHER SPECIFIED SITES	OTHER
**217**	BENIGN NEOPLASM OF BREAST	Benign
**220**	BENIGN NEOPLASM OF OVARY	Benign
**226**	BENIGN NEOPLASM OF THYROID GLANDS	Benign
**2100**	BENIGN NEOPLASM OF LIP	Benign
**2101**	BENIGN NEOPLASM OF TONGUE	Benign
**2102**	BENIGN NEOPLASM OF MAJOR SALIVARY GLANDS	Benign
**2104**	BENIGN NEOPLASM OF OTHER AND UNSPECIFIED PARTS OF MOUTH	Benign
**2105**	BENIGN NEOPLASM OF TONSIL	Benign
**2111**	BENIGN NEOPLASM OF STOMACH	Benign
**2113**	BENIGN NEOPLASM OF COLON	Benign
**2114**	BENIGN NEOPLASM OF RECTUM AND ANAL CANAL	Benign
**2115**	BENIGN NEOPLASM OF LIVER AND BILIARY PASSAGES	Benign
**2117**	BENIGN NEOPLASM OF ISLETS OF LANGERHANS	Benign
**2118**	BENIGN NEOPLASM OF RETROPERITONEUM AND PERITONEUM	Benign

**2120**	BENIGN NEOPLASM NASAL CAVITIES,MIDDLE EAR,ACCESSORY SINUSES	Benign
**2121**	BENIGN NEOPLASM OF LARYNX	Benign
**2125**	BENIGN NEOPLASM OF MEDIASTINUM	Benign
**2127**	BENIGN NEOPLASM OF HEART	Benign
**2130**	BENIGN NEOPLASM OF BONES OF SKULL AND FACE	Benign
**2131**	BENIGN NEOPLASM OF LOWER JAW BONE	Benign
**2132**	BENIGN NEOPLASM OF VERTEBRAL COLUMN,EXCLUDING SACRUM AND COCCYX	Benign
**2134**	BENIGN NEOPLASM OF SCAPULA AND LONG BONES OF UPPER LIMB	Benign
**2135**	BENIGN NEOPLASM OF SHORT BONES OF UPPER LIMB	Benign
**2136**	BENIGN NEOPLASM OF PELVIC BONES, SACRUM, AND COCCYX	Benign
**2137**	BENIGN NEOPLASM OF LONG BONES OF LOWER LIMB	Benign
**2138**	BENIGN NEOPLASM OF SHORT BONES OF LOWER LIMB	Benign
**2140**	LIPOMA OF SKIN AND SUBCUTANEOUS TISSUE OF FACE	Benign
**2141**	LIPOMA OF OTHER SKIN AND SUBCUTANEOUS TISSUE	Benign
**2148**	LIPOMA OF OTHER SPECIFIED SITES	Benign
**2149**	LIPOMA, UNSPECIFIED SITE	Benign
**2150**	BENIGN NEOPLASM OF CONNECTIVE AND SOFT TISSUE OF HEAD,FACE,NECK	Benign
**2152**	BENIGN NEOPLASM OF CONNECTIVE AND SOFT TISSUE OF UPPER LIMB (INCLUDING SHOULDER)	Benign
**2153**	BENIGN NEOPLASM OF CONNECTIVE AND SOFT TISSUE OF LOWER LIMB (HIP)	Benign
**2154**	BENIGN NEOPLASM OF CONNECTIVE AND SOFT TISSUE OF THORAX	Benign
**2156**	BENIGN NEOPLASM OF CONNECTIVE AND SOFT TISSUE OF PELVIS	Benign
**2157**	BENIGN NEOPLASM OF CONNECTIVE AND SOFT TISSUE OF TRUNK,UNSPECIFIED	Benign
**2160**	BENIGN NEOPLASM OF SKIN OF LIP	Benign
**2161**	BENIGN NEOPLASM OF EYELID, INCLUDING CANTHUS	Benign
**2162**	BENIGN NEOPLASM OF EAR AND EXTERNAL AUDITORY CANAL	Benign
**2163**	BENIGN NEOPLASM OF SKIN OF OTHER AND UNSPECIFIED PARTS OF FACE	Benign
**2164**	BENIGN NEOPLASM OF SCALP AND SKIN OF NECK	Benign
**2165**	BENIGN NEOPLASM OF SKIN OF TRUNK, EXCEPT SCROTUM	Benign
**2166**	BENIGN NEOPLASM OF SKIN OF UPPER LIMB, INCLUDING SHOULDER	Benign

**2167**	BENIGN NEOPLASM OF SKIN OF LOWER LIMB, INCLUDING HIP	Benign
**2169**	BENIGN NEOPLASM OF SKIN, SITE UNSPECIFIED	Benign
**2189**	LEIOMYOMA OF UTERUS, UNSPECIFIED	Benign
**2212**	BENIGN NEOPLASM OF VULVA	Benign
**2220**	BENIGN NEOPLASM OF TESTIS	Benign
**2221**	BENIGN NEOPLASM OF PENIS	Benign
**2230**	BENIGN NEOPLASM OF KIDNEY, EXCEPT PELVIS	Benign
**2240**	BENIGN NEOPLASM EYEBALL,EXCEPT CONJUNCTIVA,CORNEA,RETINA,CHOROID	Benign
**2241**	BENIGN NEOPLASM OF ORBIT	Benign
**2243**	BENIGN NEOPLASM OF CONJUNCTIVA	Benign
**2248**	BENIGN NEOPLASM OF OTHER SPECIFIED PARTS OF EYE	Benign
**2250**	BENIGN NEOPLASM OF BRAIN	Benign
**2251**	BENIGN NEOPLASM OF CRANIAL NERVES	Benign
**2252**	BENIGN NEOPLASM OF CEREBRAL MENINGES	Benign
**2253**	BENIGN NEOPLASM OF SPINAL CORD	Benign
**2270**	BENIGN NEOPLASM OF ADRENAL GLAND	Benign
**2271**	BENIGN NEOPLASM OF PARATHYROID GLAND	Benign
**2273**	BENIGN NEOPLASM OF PITUITARY GLAND AND CRANIOPHARYNGEAL DUCT	Benign
**2241**	BENIGN NEOPLASM OF ORBIT	Benign
**2243**	BENIGN NEOPLASM OF CONJUNCTIVA	Benign
**2298**	BENIGN NEOPLASM OF OTHER SPECIFIED SITES	Benign
**22381**	BENIGN NEOPLASM OF URETHRA	Benign
**2281**	LYMPHANGIOMA, ANY SITE	Hemangioma
**22800**	HEMANGIOMA OF UNSPECIFIED SITE	Hemangioma
**22801**	HEMANGIOMA OF SKIN AND SUBCUTANEOUS TISSUE	Hemangioma
**22802**	HEMANGIOMA OF INTRACRANIAL STRUCTURES	Hemangioma
**22804**	HEMANGIOMA OF INTRAABDOMINAL STRUCTURES	Hemangioma
**22809**	HEMANGIOMA OF OTHER SITES	Hemangioma
**2351**	NEOPLASM OF UNCERTAIN BEHAVIOR OF LIP, ORAL CAVITY, AND PHARYNX	Nos

**2352**	NEOPLASM OF UNCERTAIN BEHAVIOR OF STOMACH,INTESTINES,RECTUM	Nos
**2354**	NEOPLASM OF UNCERTAIN BEHAVIOR,RETROPERITONEUM,PERITONEUM	Nos
**2357**	NEOPLASM OF UNCERTAIN BEHAVIOR OF TRACHEA, BRONCHUS, AND LUNG	Nos
**2364**	NEOPLASM OF UNCERTAIN BEHAVIOR OF TESTIS	Nos
**2369**	NEOPLASM OF UNCERTAIN BEHAVIOR OF UNSPECIFIED URINARY ORGANS	Nos
**2370**	NEOPLASM UNCERTAIN BEHAVIOR,PITUITARY GLAND,CRANIOPHARYNGEAL DUCT	Nos
**2380**	NEOPLASM OF UNCERTAIN BEHAVIOR OF BONE AND ARTICULAR CARTILAGE	Nos
**2381**	NEOPLASM OF UNCERTAIN BEHAVIOR,CONNECTIVE AND SOFT TISSUE	Nos
**2382**	NEOPLASM OF UNCERTAIN BEHAVIOR OF SKIN	Nos
**2387**	NEOPLASM UNCERTAIN BEHAVIOR OF LYMPHATIC AND HEMATOPOIETIC TISSUE	Nos
**2387**	NEOPLASM UNCERTAIN BEHAVIOR OF LYMPHATIC AND HEMATOPOIETIC TISSUE	Nos
**2388**	NEOPLASM OF UNCERTAIN BEHAVIOR OF OTHER SPECIFIED SITES	Nos
**23691**	NEOPLASM OF UNCERTAIN BEHAVIOR OF KIDNEY AND URETER	Nos
**23879**	NEOPLASM OF UNCERTAIN BEHAVIOU OF OTHER LYMPHATIC AND HEMATOPOIETIC TISSUE	Nos
